# 4,4,5,5-Tetra­methyl-2-(3,4,5-trimethoxy­phen­yl)imidazolidine-1-oxyl 3-oxide

**DOI:** 10.1107/S1600536809029274

**Published:** 2009-08-08

**Authors:** Hai-Bo Wang, Lin-Lin Jing, Peng Gao, Xiao-Li Sun

**Affiliations:** a Department of Chemistry, School of Pharmacy, Fourth Military Medical University, Changle West Road 17, 710032 Xi-An, People’s Republic of China

## Abstract

In the title nitronyl nitroxide radical compound, C_16_C_23_N_2_O_5_, the imidazole and benzene rings are twisted with respect to each other, making a dihedral angle of 26.2 (4)°. The imidazole ring adopts a half-chair conformation. Weak C—H⋯π inter­actions are also found.

## Related literature

For the preparation of the title compound see: Ullman *et al.* (1974 ▶). For related structures, see: Feher *et al.* (2008 ▶); Gao *et al.* (2009 ▶); Qin *et al.* (2009 ▶); Cirujeda *et al.* (1995 ▶); Matsushita *et al.* (1997 ▶). For the coordination properties of the title compound and its use in the formation of mol­ecule-based magnetic materials, see: Takui *et al.* (2009 ▶). For puckering parameters, see: Cremer & Pople (1975 ▶).
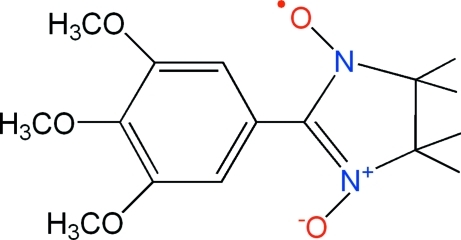


## Experimental

### 

#### Crystal data

C_16_H_23_N_2_O_5_
*M* *_r_* = 323.36
Orthorhombic, 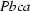

*a* = 20.623 (3) Å
*b* = 7.2168 (12) Å
*c* = 22.831 (4) Å
*V* = 3398.0 (10) Å^3^
*Z* = 8
Mo *K*α radiation
μ = 0.09 mm^−1^
*T* = 296 K
0.32 × 0.25 × 0.17 mm

#### Data collection

Bruker APEXII diffractometer
Absorption correction: none
15858 measured reflections
3027 independent reflections
1519 reflections with *I* > 2σ(*I*)
*R*_int_ = 0.062

#### Refinement

*R*[*F* ^2^ > 2σ(*F* ^2^)] = 0.046
*wR*(*F* ^2^) = 0.119
*S* = 1.06
3027 reflections
216 parameters
H-atom parameters constrained
Δρ_max_ = 0.17 e Å^−3^
Δρ_min_ = −0.23 e Å^−3^

### 

Data collection: *APEX2* (Bruker, 2007 ▶); cell refinement: *SAINT* (Bruker, 2007 ▶); data reduction: *SAINT*; program(s) used to solve structure: *SHELXS97* (Sheldrick, 2008 ▶); program(s) used to refine structure: *SHELXL97* (Sheldrick, 2008 ▶); molecular graphics: *ORTEPIII* (Burnett & Johnson, 1996 ▶) and *ORTEP-3 for Windows* (Farrugia, 1997 ▶); software used to prepare material for publication: *SHELXTL* (Sheldrick, 2008 ▶).

## Supplementary Material

Crystal structure: contains datablocks I, global. DOI: 10.1107/S1600536809029274/dn2472sup1.cif

Structure factors: contains datablocks I. DOI: 10.1107/S1600536809029274/dn2472Isup2.hkl

Additional supplementary materials:  crystallographic information; 3D view; checkCIF report

## Figures and Tables

**Table 1:** Hydrogen-bond geometry (Å, °)

*D*—H⋯*A*	*D*—H	H⋯*A*	*D*⋯*A*	*D*—H⋯*A*
C14—H14*A*⋯*Cg*2^i^	0.96	2.80	3.644 (2)	147

Symmetry code: (i) 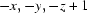. *Cg*2 is the centroid of the phenyl ring.

**Fig. 1.**
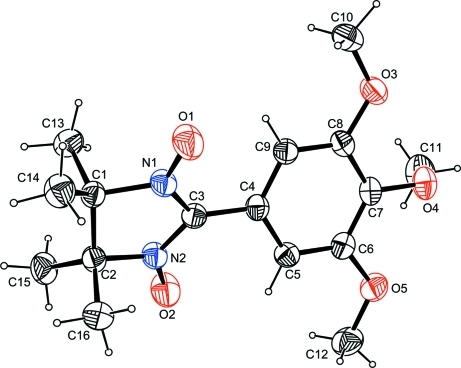
Molecular structure of the title compound (I), showing the atom labeling scheme. Displacement ellipsoids are drawn at the 30% probability level.

## supplementary crystallographic information

### Comment

The title radical compound was obtained from the oxidation of 4,4,5,5- tetramethyl-2-(3,4,5-trimethoxybenzenyl)-imidazolidine-1,3-diol, which was prepared by the condensation of 3,4,5-trimethoxybenzaldehyde with 2,3-Dimethyl-2,3-bis(hydroxyl-amino)butane. The title compound was used for coordination with many metal cations, such as Mn^2+^, Cu^2+^, and Ni^2+^, in order to form some molecule-based magnetic materials (Takui *et al.*, 2009).

The molecular structure of the title compound is shown in Fig 1. Examination of bond length within the five membered rings shows an average structure as observed with related compounds (Cirujeda *et al.*, 1995; Feher *et al.*, 2008; Gao *et al.*, 2009; Matsushita *et al.*, 1997; Qin *et al.*, 2009).

The imidazole and the phenyl rings are twisted with respect to each other making a dihedral angle of 26.2 (4)°. The imidazole ring has an half-chair conformation with puckering parameters O(2)=. 0.0275 (2)Å and φ= 233.0 (5)°(Cremer & Pople, 1975). The crystal structure is stabilized by weak C—H···π (Table 1, *Cg*2 is the centroid of the phenyl ring) and van der Waals interactions.

### Experimental

The compound 4,4,5,5-tetramethyl-2-(3,4,5-trimethoxybenzenyl)-imidazolidine -1-oxyl-3-oxide was prepared according to the method reported by Ullman *et al.* (1974). 2,3-Dimethyl-2,3-bis(hydroxylamino) butane (1.48 g, 10.0 mmol) and 3,4,5-trimethoxybenzaldehyde (1.96 g, 10.0 mmol) were dissolved in a methanol-water mixture (2:1), which was stirred for 5 h at reflux temperature, then cooled to room temperature and filtered. The white powder was washed by methanol. This product was dried under vaccum, then, it was suspended in dichloromethane (50.0 ml) and the water solution (30.0 ml) of NaIO_4 _(1.7 g) was added and stirred at ice bath for 20 min. The reaction mixture was extracted by dichloromethane (30.0 ml) for twice and the organic layer was combined and dried over Na_2_SO_4_. Then the solvent was removed to give a dark blue residue which was purified by a flash column chromatography (eluent, ether and petroleum ether, the ratio of volume is 2 to 1) to yield the title compound (I) as a dark blue powder. Single crystals of compound (I) were obtained from the mixed solution of *n*-heptane and dichloromethane (the ratio of volume is 1 to 1).

### Refinement

All H atoms were fixed geometrically and treated as riding with C—H = 0.96 Å (methyl) or 0.93 Å (aromatic) with *U*_iso_(H) = 1.2*U*_eq_(C_aromatic_) or *U*_iso_(H) = 1.5*U*_eq_(C_methyl_).

### Crystal data

**Table:**

C_16_H_23_N_2_O_5_	*F*(000) = 1384
*M**_r_* = 323.36	*D*_x_ = 1.264 Mg m^−^^3^
Orthorhombic, *P**b**c**a*	Mo *K*α radiation, λ = 0.71073 Å
Hall symbol: -P 2ac 2ab	Cell parameters from 1322 reflections
*a* = 20.623 (3) Å	θ = 3.1–18.2°
*b* = 7.2168 (12) Å	µ = 0.09 mm^−^^1^
*c* = 22.831 (4) Å	*T* = 296 K
*V* = 3398.0 (10) Å^3^	Block, blue
*Z* = 8	0.32 × 0.25 × 0.17 mm

### Data collection

**Table:**

Bruker SMART APEX2 diffractometer	1519 reflections with *I* > 2σ(*I*)
Radiation source: fine-focus sealed tube	*R*_int_ = 0.062
graphite	θ_max_ = 25.1°, θ_min_ = 1.8°
Detector resolution: 0 pixels mm^-1^	*h* = −12→24
φ and ω scans	*k* = −8→8
15858 measured reflections	*l* = −27→26
3027 independent reflections	

### Refinement

**Table:**

Refinement on *F*^2^	Secondary atom site location: difference Fourier map
Least-squares matrix: full	Hydrogen site location: inferred from neighbouring sites
*R*[*F*^2^ > 2σ(*F*^2^)] = 0.046	H-atom parameters constrained
*wR*(*F*^2^) = 0.119	*w* = 1/[σ^2^(*F*_o_^2^) + (0.05*P*)^2^] where *P* = (*F*_o_^2^ + 2*F*_c_^2^)/3
*S* = 1.06	(Δ/σ)_max_ = 0.001
3027 reflections	Δρ_max_ = 0.17 e Å^−^^3^
216 parameters	Δρ_min_ = −0.23 e Å^−^^3^
0 restraints	Extinction correction: *SHELXL97* (Sheldrick, 2008), Fc^*^=kFc[1+0.001xFc^2^λ^3^/sin(2θ)]^-1/4^
Primary atom site location: structure-invariant direct methods	Extinction coefficient: 0.0019 (5)

### Special details

**Table:**

Geometry. All e.s.d.'s (except the e.s.d. in the dihedral angle between two l.s. planes) are estimated using the full covariance matrix. The cell e.s.d.'s are taken into account individually in the estimation of e.s.d.'s in distances, angles and torsion angles; correlations between e.s.d.'s in cell parameters are only used when they are defined by crystal symmetry. An approximate (isotropic) treatment of cell e.s.d.'s is used for estimating e.s.d.'s involving l.s. planes.
Refinement. Refinement of *F*^2^ against ALL reflections. The weighted *R*-factor *wR* and goodness of fit *S* are based on *F*^2^, conventional *R*-factors *R* are based on *F*, with *F* set to zero for negative *F*^2^. The threshold expression of *F*^2^ > σ(*F*^2^) is used only for calculating *R*-factors(gt) *etc*. and is not relevant to the choice of reflections for refinement. *R*-factors based on *F*^2^ are statistically about twice as large as those based on *F*, and *R*- factors based on ALL data will be even larger.

### Fractional atomic coordinates and isotropic or equivalent isotropic displacement parameters (Å^2^)

**Table:**

	*x*	*y*	*z*	*U*_iso_*/*U*_eq_	
N1	0.06793 (9)	0.2073 (3)	0.49665 (8)	0.0570 (5)	
N2	0.15280 (9)	0.0432 (3)	0.47376 (8)	0.0576 (5)	
O1	0.01712 (9)	0.2652 (3)	0.52296 (7)	0.0913 (6)	
O2	0.20243 (8)	−0.0630 (3)	0.47707 (7)	0.0903 (6)	
O3	0.07153 (8)	0.1394 (2)	0.72841 (6)	0.0717 (5)	
O4	0.12645 (7)	−0.1894 (2)	0.74689 (6)	0.0720 (5)	
O5	0.17240 (8)	−0.3947 (2)	0.65885 (7)	0.0743 (5)	
C1	0.08745 (10)	0.2830 (3)	0.43780 (9)	0.0511 (6)	
C2	0.13132 (10)	0.1227 (3)	0.41649 (9)	0.0548 (6)	
C3	0.11082 (10)	0.0839 (3)	0.51744 (9)	0.0506 (6)	
C4	0.11306 (10)	0.0103 (3)	0.57690 (9)	0.0505 (6)	
C5	0.14140 (10)	−0.1615 (3)	0.58697 (10)	0.0533 (6)	
H5	0.1574	−0.2313	0.5559	0.064*	
C6	0.14551 (10)	−0.2276 (3)	0.64375 (10)	0.0550 (6)	
C7	0.12210 (10)	−0.1231 (3)	0.69035 (10)	0.0542 (6)	
C8	0.09320 (11)	0.0479 (3)	0.67984 (9)	0.0543 (6)	
C9	0.08840 (10)	0.1132 (3)	0.62324 (9)	0.0539 (6)	
H9	0.0686	0.2266	0.6161	0.065*	
C10	0.03918 (13)	0.3130 (4)	0.71996 (10)	0.0823 (8)	
H10A	0.0693	0.4022	0.7047	0.123*	
H10B	0.0224	0.3562	0.7567	0.123*	
H10C	0.0041	0.2971	0.6928	0.123*	
C11	0.18747 (13)	−0.1598 (5)	0.77318 (11)	0.1017 (10)	
H11A	0.2203	−0.2234	0.7511	0.153*	
H11B	0.1868	−0.2064	0.8126	0.153*	
H11C	0.1968	−0.0295	0.7737	0.153*	
C12	0.19952 (13)	−0.5035 (4)	0.61337 (12)	0.0869 (8)	
H12A	0.1663	−0.5354	0.5857	0.130*	
H12B	0.2177	−0.6146	0.6296	0.130*	
H12C	0.2329	−0.4343	0.5940	0.130*	
C13	0.12386 (12)	0.4629 (3)	0.45043 (11)	0.0726 (7)	
H13A	0.0965	0.5449	0.4724	0.109*	
H13B	0.1358	0.5209	0.4141	0.109*	
H13C	0.1622	0.4360	0.4727	0.109*	
C14	0.02814 (11)	0.3224 (3)	0.40075 (10)	0.0722 (7)	
H14A	0.0019	0.2129	0.3984	0.108*	
H14B	0.0414	0.3586	0.3621	0.108*	
H14C	0.0035	0.4207	0.4183	0.108*	
C15	0.18983 (12)	0.1816 (4)	0.38065 (10)	0.0783 (8)	
H15A	0.2168	0.2619	0.4037	0.117*	
H15B	0.1756	0.2462	0.3462	0.117*	
H15C	0.2141	0.0739	0.3694	0.117*	
C16	0.09402 (13)	−0.0316 (3)	0.38572 (10)	0.0765 (8)	
H16A	0.1214	−0.1385	0.3818	0.115*	
H16B	0.0808	0.0099	0.3476	0.115*	
H16C	0.0564	−0.0633	0.4084	0.115*	

### Atomic displacement parameters (Å^2^)

**Table:**

	*U*^11^	*U*^22^	*U*^33^	*U*^12^	*U*^13^	*U*^23^
N1	0.0488 (12)	0.0639 (13)	0.0583 (12)	0.0123 (10)	0.0125 (10)	0.0033 (10)
N2	0.0509 (11)	0.0636 (13)	0.0583 (13)	0.0127 (11)	0.0095 (10)	0.0047 (10)
O1	0.0890 (14)	0.1057 (15)	0.0792 (11)	0.0366 (11)	0.0233 (11)	0.0175 (10)
O2	0.0745 (12)	0.1091 (15)	0.0872 (12)	0.0401 (11)	0.0215 (10)	0.0192 (10)
O3	0.0827 (13)	0.0798 (12)	0.0526 (10)	0.0157 (10)	0.0074 (8)	−0.0006 (9)
O4	0.0655 (12)	0.0913 (13)	0.0591 (11)	−0.0002 (9)	−0.0019 (9)	0.0204 (9)
O5	0.0798 (12)	0.0648 (12)	0.0783 (11)	0.0196 (10)	0.0001 (10)	0.0135 (10)
C1	0.0527 (14)	0.0518 (15)	0.0487 (13)	0.0028 (12)	0.0001 (11)	0.0055 (11)
C2	0.0590 (15)	0.0580 (15)	0.0474 (13)	0.0022 (12)	0.0058 (11)	0.0036 (12)
C3	0.0454 (13)	0.0516 (14)	0.0548 (14)	0.0060 (12)	0.0035 (12)	0.0021 (11)
C4	0.0450 (13)	0.0560 (15)	0.0504 (14)	−0.0007 (12)	0.0011 (11)	0.0049 (12)
C5	0.0475 (14)	0.0561 (16)	0.0561 (15)	0.0046 (11)	0.0013 (11)	−0.0007 (12)
C6	0.0453 (14)	0.0575 (16)	0.0623 (16)	0.0001 (12)	−0.0007 (12)	0.0082 (13)
C7	0.0478 (14)	0.0651 (17)	0.0496 (14)	−0.0037 (13)	0.0000 (11)	0.0112 (13)
C8	0.0517 (14)	0.0636 (16)	0.0474 (15)	−0.0029 (13)	0.0051 (11)	−0.0016 (12)
C9	0.0533 (14)	0.0544 (14)	0.0539 (15)	0.0038 (12)	0.0032 (11)	0.0032 (12)
C10	0.096 (2)	0.081 (2)	0.0701 (16)	0.0165 (17)	0.0143 (15)	−0.0100 (15)
C11	0.089 (2)	0.144 (3)	0.0727 (17)	−0.012 (2)	−0.0241 (17)	0.0182 (18)
C12	0.087 (2)	0.0655 (18)	0.108 (2)	0.0200 (15)	0.0072 (18)	0.0042 (16)
C13	0.0812 (19)	0.0569 (16)	0.0797 (17)	−0.0028 (14)	0.0029 (14)	0.0004 (13)
C14	0.0669 (17)	0.0819 (19)	0.0679 (15)	0.0090 (14)	−0.0072 (13)	0.0070 (14)
C15	0.0740 (19)	0.087 (2)	0.0736 (16)	0.0027 (15)	0.0289 (14)	0.0119 (14)
C16	0.097 (2)	0.0662 (17)	0.0662 (16)	−0.0037 (15)	−0.0008 (15)	−0.0077 (13)

### Geometric parameters (Å, °)

**Table:**

N1—O1	1.278 (2)	C8—C9	1.379 (3)
N1—C3	1.342 (2)	C9—H9	0.9300
N1—C1	1.506 (3)	C10—H10A	0.9600
N2—O2	1.281 (2)	C10—H10B	0.9600
N2—C3	1.353 (2)	C10—H10C	0.9600
N2—C2	1.495 (3)	C11—H11A	0.9600
O3—C8	1.366 (2)	C11—H11B	0.9600
O3—C10	1.433 (3)	C11—H11C	0.9600
O4—C7	1.380 (2)	C12—H12A	0.9600
O4—C11	1.410 (3)	C12—H12B	0.9600
O5—C6	1.372 (3)	C12—H12C	0.9600
O5—C12	1.417 (3)	C13—H13A	0.9600
C1—C14	1.514 (3)	C13—H13B	0.9600
C1—C13	1.527 (3)	C13—H13C	0.9600
C1—C2	1.547 (3)	C14—H14A	0.9600
C2—C15	1.519 (3)	C14—H14B	0.9600
C2—C16	1.525 (3)	C14—H14C	0.9600
C3—C4	1.459 (3)	C15—H15A	0.9600
C4—C9	1.389 (3)	C15—H15B	0.9600
C4—C5	1.390 (3)	C15—H15C	0.9600
C5—C6	1.384 (3)	C16—H16A	0.9600
C5—H5	0.9300	C16—H16B	0.9600
C6—C7	1.391 (3)	C16—H16C	0.9600
C7—C8	1.391 (3)		
			
O1—N1—C3	126.26 (18)	O3—C10—H10A	109.5
O1—N1—C1	121.31 (18)	O3—C10—H10B	109.5
C3—N1—C1	112.36 (17)	H10A—C10—H10B	109.5
O2—N2—C3	126.70 (19)	O3—C10—H10C	109.5
O2—N2—C2	121.20 (17)	H10A—C10—H10C	109.5
C3—N2—C2	111.83 (17)	H10B—C10—H10C	109.5
C8—O3—C10	117.76 (17)	O4—C11—H11A	109.5
C7—O4—C11	113.82 (18)	O4—C11—H11B	109.5
C6—O5—C12	117.56 (18)	H11A—C11—H11B	109.5
N1—C1—C14	110.53 (17)	O4—C11—H11C	109.5
N1—C1—C13	105.77 (17)	H11A—C11—H11C	109.5
C14—C1—C13	110.08 (19)	H11B—C11—H11C	109.5
N1—C1—C2	99.52 (16)	O5—C12—H12A	109.5
C14—C1—C2	115.92 (18)	O5—C12—H12B	109.5
C13—C1—C2	114.05 (18)	H12A—C12—H12B	109.5
N2—C2—C15	110.07 (18)	O5—C12—H12C	109.5
N2—C2—C16	105.78 (18)	H12A—C12—H12C	109.5
C15—C2—C16	110.91 (19)	H12B—C12—H12C	109.5
N2—C2—C1	100.67 (16)	C1—C13—H13A	109.5
C15—C2—C1	115.15 (19)	C1—C13—H13B	109.5
C16—C2—C1	113.33 (18)	H13A—C13—H13B	109.5
N1—C3—N2	107.77 (18)	C1—C13—H13C	109.5
N1—C3—C4	126.3 (2)	H13A—C13—H13C	109.5
N2—C3—C4	125.9 (2)	H13B—C13—H13C	109.5
C9—C4—C5	120.32 (19)	C1—C14—H14A	109.5
C9—C4—C3	120.2 (2)	C1—C14—H14B	109.5
C5—C4—C3	119.5 (2)	H14A—C14—H14B	109.5
C6—C5—C4	119.2 (2)	C1—C14—H14C	109.5
C6—C5—H5	120.4	H14A—C14—H14C	109.5
C4—C5—H5	120.4	H14B—C14—H14C	109.5
O5—C6—C5	124.3 (2)	C2—C15—H15A	109.5
O5—C6—C7	115.1 (2)	C2—C15—H15B	109.5
C5—C6—C7	120.6 (2)	H15A—C15—H15B	109.5
O4—C7—C6	120.4 (2)	C2—C15—H15C	109.5
O4—C7—C8	119.8 (2)	H15A—C15—H15C	109.5
C6—C7—C8	119.9 (2)	H15B—C15—H15C	109.5
O3—C8—C9	124.9 (2)	C2—C16—H16A	109.5
O3—C8—C7	115.40 (19)	C2—C16—H16B	109.5
C9—C8—C7	119.7 (2)	H16A—C16—H16B	109.5
C8—C9—C4	120.3 (2)	C2—C16—H16C	109.5
C8—C9—H9	119.8	H16A—C16—H16C	109.5
C4—C9—H9	119.8	H16B—C16—H16C	109.5
			
O1—N1—C1—C14	−36.5 (3)	C2—N2—C3—C4	172.4 (2)
C3—N1—C1—C14	146.39 (19)	N1—C3—C4—C9	−26.4 (3)
O1—N1—C1—C13	82.7 (2)	N2—C3—C4—C9	151.8 (2)
C3—N1—C1—C13	−94.5 (2)	N1—C3—C4—C5	155.1 (2)
O1—N1—C1—C2	−158.87 (19)	N2—C3—C4—C5	−26.7 (3)
C3—N1—C1—C2	24.0 (2)	C9—C4—C5—C6	−0.9 (3)
O2—N2—C2—C15	−40.2 (3)	C3—C4—C5—C6	177.65 (19)
C3—N2—C2—C15	145.36 (19)	C12—O5—C6—C5	2.1 (3)
O2—N2—C2—C16	79.7 (2)	C12—O5—C6—C7	−177.4 (2)
C3—N2—C2—C16	−94.8 (2)	C4—C5—C6—O5	−179.98 (19)
O2—N2—C2—C1	−162.2 (2)	C4—C5—C6—C7	−0.5 (3)
C3—N2—C2—C1	23.4 (2)	C11—O4—C7—C6	82.3 (3)
N1—C1—C2—N2	−25.95 (19)	C11—O4—C7—C8	−98.7 (3)
C14—C1—C2—N2	−144.43 (19)	O5—C6—C7—O4	−0.3 (3)
C13—C1—C2—N2	86.2 (2)	C5—C6—C7—O4	−179.84 (19)
N1—C1—C2—C15	−144.27 (19)	O5—C6—C7—C8	−179.30 (19)
C14—C1—C2—C15	97.3 (2)	C5—C6—C7—C8	1.2 (3)
C13—C1—C2—C15	−32.1 (3)	C10—O3—C8—C9	2.5 (3)
N1—C1—C2—C16	86.5 (2)	C10—O3—C8—C7	−177.7 (2)
C14—C1—C2—C16	−31.9 (3)	O4—C7—C8—O3	0.7 (3)
C13—C1—C2—C16	−161.33 (18)	C6—C7—C8—O3	179.72 (19)
O1—N1—C3—N2	172.6 (2)	O4—C7—C8—C9	−179.5 (2)
C1—N1—C3—N2	−10.4 (2)	C6—C7—C8—C9	−0.5 (3)
O1—N1—C3—C4	−8.9 (4)	O3—C8—C9—C4	178.9 (2)
C1—N1—C3—C4	168.07 (19)	C7—C8—C9—C4	−0.9 (3)
O2—N2—C3—N1	176.8 (2)	C5—C4—C9—C8	1.6 (3)
C2—N2—C3—N1	−9.1 (2)	C3—C4—C9—C8	−176.9 (2)
O2—N2—C3—C4	−1.7 (4)		

### Hydrogen-bond geometry (Å, °)

**Table:**

*D*—H···*A*	*D*—H	H···*A*	*D*···*A*	*D*—H···*A*
C14—H14A···Cg2^i^	0.96	2.80	3.644 (2)	147

Symmetry codes: (i) −*x*, −*y*, −*z*+1.
